# Prevalence and associated factors of possible sarcopenia and sarcopenia: findings from a Chinese community-dwelling old adults cross-sectional study

**DOI:** 10.1186/s12877-022-03286-y

**Published:** 2022-07-18

**Authors:** Jiazhi Wang, Changge Liu, Lin Zhang, Ning Liu, Lei Wang, Jingqiong Wu, Yizhao Wang, Huimin Hao, Longjun Cao, Shilei Yuan, Liping Huang

**Affiliations:** 1 Tianjin University of Sport, No.16 Donghai Road, West Tuanbo New Town, Jinghai District, Tianjin, P.R. China; 2 Tianjin Yanan Hospital, No. 45 Yashi Road, Nankai District, Tianjin, P.R. China

**Keywords:** Possible sarcopenia, Sarcopenia, Physical activity, Body fat, Associated factor

## Abstract

**Purpose:**

To describe the prevalence and analyse the associated factors of possible sarcopenia and sarcopenia among community-dwelling old adults in China, in order to provide effective strategies for early prevention and treatment of sarcopenia.

**Methods:**

This cross-sectional study evaluated community-dwelling old adults aged over 60 years. The basic information, morphological indices, body composition, physical activities were collected and assessed. Possible sarcopenia and sarcopenia were diagnosed by the criteria of Asian Working Group for Sarcopenia (AWGS) in 2019. A multivariate logistic regression model with stepwise method was employed to identify factors associated with possible sarcopenia and sarcopenia.

**Results:**

In total 729 old adults from Tianjin were included in this study. Eighty-one participants were diagnosed with possible sarcopenia (prevalence of 11.11%). Seventy-five participants were diagnosed with sarcopenia (prevalence of 10.29%). Age (odds ratio (OR):1.047, 95% confidence interval (CI): 1.055–1.090) and lower physical activities (low level OR:4.171, 95% CI:1.790–9.720; medium level OR:2.634, 95% CI:1.352–5.132) were significantly associated with possible sarcopenia. Age (OR:1.187, 95% CI:1.124–1.253), higher body fat percentage (OR:1.225, 95% CI:1.140–1.317), lower BMI (OR:0.424, 95% CI:0.346–0.519), lower mini-mental state examination (MMSE) scores (OR:0.865,95% CI:0.781–0.958) and low physical activities (OR:4.638, 95% CI:1.683–12.782) were significantly associated with sarcopenia.

**Conclusion:**

Possible sarcopenia and sarcopenia are prevalent among community-dwelling old adults in China. Ageing and lower physical activities were both associated with possible sarcopenia and sarcopenia. Old adults with sarcopenia more likely have higher body fat percentage, lower BMI and lower cognitive function compared with those without this condition.

## Introduction

Sarcopenia is a geriatric syndrome characterized by loss of skeletal muscle mass, strength and function [1, 2], and is associated with cardiovascular disease, diabetes mellitus, and cognitive impairment. This condition is closely related decreased motor function, resulting in an increased risk of disability, loss of independence, and mortality [3, 4]. In 2019, the Asia Working Group of Sarcopenia (AWGS) adjusted the diagnostic criteria of sarcopenia, and put forward a new concept, possible sarcopenia, which means lower grip strength but within the normal range of skeletal muscle mass. Possible sarcopenia can be used for screening and early identifying the risk of sarcopenia to prevent sarcopenia [5]. The concept of possible sarcopenia was proposed based on recent studies found that grip strength was more associated with disability, cardiovascular disease incidence and all-cause mortality than muscle mass [6–8]. Primary sarcopenia is not easy to be realized. When old adults have sarcopenia, their physical functions have been greatly impaired, and the risk of disability and weakness is increased [9]. Therefore, early identifying sarcopenia and associated factors and taking intervention measures can effectively prevent the adverse health outcomes such as dysfunction, disability, frailty, etc. According to the AWGS2019 criterion, the incidence of possible sarcopenia in old adults with different ages and regions were reported differently in China, Japan and South Korea, ranging from 2.9 to 38.5% [10–12], which may be related to the age, sex, life and eating habits of the subjects. Wu et al. reported that the prevalence of possible sarcopenia among over 60 years old adults in Chinese urban is 31.1%, which come from the comprehensive results of many urban populations. We still need to know the prevalence of possible sarcopenia in megalopolitan communities in order to early prevent sarcopenia and its adverse health consequences.

Studies have reported that the associated factors of sarcopenia included ageing, diabetes mellitus, low physical activities, etc., while high BMI can reduce the risk of sarcopenia [13, 14]. Realizing the associated factors of sarcopenia and its early manifestations is also of great significance to prevent sarcopenia. A study of community-dwelling old adults over the age of 65 by Miura et al. found that low BMI and low physical activity level were associated with possible sarcopenia [11], while Wu et al. found that aging, falls and chronic diseases increased the risk of possible sarcopenia [10]. However, there is still something need to be studied or improved. Participants aged 60–94 years were recruited in Xin Wu 2021 article while appendicular skeletal muscle mass (ASM) is calculated using an anthropometric equation which was established by including participants aged 18–69 years [10, 15], the estimation of ASM was hard to avoid deviation. In addition, AWGS2019 recommend using dual-energy X-ray absorptiometry (DXA) or bioelectrical impedance analysis (BIA) to measure ASM [5]. Equation estimation of ASM could be less precise than direct measurement with some error rate. More importantly, the factors that might be associated to possible sarcopenia and sarcopenia, including education level, traffic habits, physical activity level, sleep status and nutritional factors like dietary habit and defecation situation should be considered. By analysing the associated factors of possible sarcopenia and sarcopenia at the same time, we can find out the associated factors before and after the occurrence of the disease, and the results may be helpful for the prevention and treatment of sarcopenia. The reports on the associated factors of sarcopenia from the literatures were different, and further analysis is needed. Therefore, our study aims to analyse the prevalence and associated factors of probable sarcopenia and sarcopenia among old adults in the metropolis community by means of multivariate stepwise logistic regression, in order to provide research basis for early screening, prevention and treatment of sarcopenia.

## Methods

### Participants

Participants aged 60 years and older were eventually recruited in this study, all participants were community-dwelling old adults that received medical examinations in Tianjin Yan’an Hospital from September 2017 to January 2019. This study was approved by the Human Ethics Committees of Tianjin University of Sport, which also conformed to provisions of the Declaration of Helsinki. All participants signed the informed consent. The participants in the unstable period of serious diseases such as cardiovascular and cerebrovascular disease, lung diseases, liver diseases, cancer, and fracture were excluded during the participants screening.

### Demographic information

Basic information questionnaires included age, sex, disease history, education background, living habit such as diet and main mean of transport, smoking history, and drinking history. The information was based on doctors’ diagnosis of self-reports. Participants were asked if they had been diagnosed with conditions listed below by a doctor (yes or no): hyperlipidemia, diabetes mellitus, arthritis, osteoporosis. Information on smoking and drinking was based on self-reports. Smoking status (“Have you ever chewed tobacco?” The answer was “yes” or “no.”). Frequency of alcohol consumption (“How often do you drink alcohol?” The answer was “more than 3 times a week” or “1-3 times a week” or “no”). Education background (“What is your education background?” The answer was “primary school or below” or “secondary school or technical secondary school” or “university, college or above”). Dietary habit question 1 (“What main type of staple food do you eat?” The answer was “refined grain” or “coarse food grain” or “refined grain and coarse food grain are equal”). Dietary habit question 2 (“Do you have the habit of drinking milk?” The answer was “every day” or “4-6 times a week” or “1–3 times a week” or “no”). Defecation (“How are your bowel movements?” The answer was “at least once a day” or “once every two days or above”). Means of transport (“What is your daily mean of transport?” The answer was “Mainly by walking and riding bikes and other relatively high-intensity means of transport” or “Mainly by driving and taking the subway and other relatively low-intensity means of transport”). Morphological indices included height, weight and blood pressure. Height (m) and weight (kg) were measured using an electronic height meter (Jianmin RG-2, China) and an electronic scale (Jianmin RCS-2, China). Body mass index (BMI) is defined as weight divided by height squared (kg/m^2^). Blood pressure (mmHg) was measured with an electronic sphygmomanometer (OMRON-HBP-9020, Japan). Participants were diagnosed with high blood pressure according to 2019 Chinese guidelines on the management of hypertension in the elderly (Old adults with systolic pressure ≥ 140 mmHg or/and diastolic pressure ≥ 90 mmHg defined as hypertension).

### Health status and body composition

Mini-mental state examination (MMSE) was used to assess the cognitive function of the old adults [16]. International Physical Activity Questionnaire long form was used to assess the sedentary time and physical activity level [17]. Tables 1 and 2 show the physical activity attributes, their assigned values and the criteria of physical activity levels, respectively. Total energy expenditure (MET·min/week) for the past 7 days were calculated and the physical activity levels (low, moderate or high level) of participants were determined according to activities energy expenditure criteria. The Pittsburgh Sleep Quality Index (PSQI) was used to assess sleep quality [18]. Body composition analyser with BIA (InBODY570, South Korea) was used to measure ASM (kg) and body fat percentage (fat%). The ASM index (ASMI) was defined as ASM divided by height squared (kg/m^2^). The grip strength (kg) was measured with an electronic grip dynamometer (WCS-100, China). During the test, the participants stood naturally and held the grip dynamometer with their dominant hands, and the upper limb extended at an angle of 30° from the body. Three times measurement was done for everyone, and the maximum value recorded as the grip strength. Gait speed (m/s) was measured with the time costed for the participants to walk 6 m at their normal speed. All test and questionnaires are conducted by systematically trained medical personnel.

**Table 1:** Physical activity type and their energy expenditure

Type of physical activity	Physical activity program	Physical activity intensity	Assigned value (MET)
Work- related	Walk	Walk	3.3
	Moderate intensity	Moderate	4.0
	High intensity	High	8.8
Transport-related	Walk	Walk	3.3
	Bicycling	Moderate	6.0
Household-and-gardening-related	Moderate intensity indoor housework	Moderate	3.0
	Moderate intensity outdoor housework	Moderate	4.0
	High intensity outdoor housework	Moderate	5.5
Leisure-related	Walk	Walk	3.3
	Moderate intensity	Moderate	4.0
	High intensity	High	8.0

**Table 2:** The criteria of physical activity levels

Grouping	Criteria
High physical activity	Meet either of the following 2 criteria
	1 Have all kinds of high intensity physical activities ≥3 days, and total weekly physical activity level ≥ 1500MET·min/week.
	2 Have physical activities ≥7 days, and total weekly physical activity level ≥ 3000MET·min/week.
Moderate physical activity	Meet either of the following 3 criteria
	1 At least 20 minutes of high intensity physical activity each day.
	2 At least 30 minutes of moderate intensity physical activity and/or walking each day.
	3 Have physical activities ≥5 days, and total weekly physical activity level ≥ 600MET·min/week.
Low physical activity	Meet either of the following 2 criteria
	1 No activity was reported.
	2 Some activities were reported but did not meet the above criteria of high and moderate physical activity.

### Assessment of possible sarcopenia, sarcopenia

According to the AWGS2019 diagnostic methods and criteria, we used ASMI to assess muscle mass, grip strength to assess muscle strength, and gait speed to assess physical performance. From AWGS to AWGS2019, and from EWGSOP to EWGSOP2, these methods are always recommended, and other methods were added or subtracted [5, 19–21]. Possible sarcopenia is defined by low muscle strength with or without reduced physical performance (male ASMI≥7.0 kg/m^2^, grip strength<28 kg; female ASMI ≥5.7 kg/m^2^, grip strength<18 kg). The key diagnosis criteria of possible sarcopenia according to AWGS2019 is low grip strength whatever physical performance is normal or reduced. To distinguish possible sarcopenia from sarcopenia, we chose low grip strength without reduced muscle mass as the definition of possible sarcopenia. Sarcopenia is diagnosed when low muscle mass plus low muscle strength or/and low physical performance are detected (male ASMI<7.0 kg/m^2^, grip strength<28 kg or/and gait speed<1 m/s; female ASMI<5.7 kg/m^2^, grip strength<18 kg or/and gait speed<1 m/s). Other participants without any low muscle mass, low muscle strength and low physical performance were classified as “no sarcopenia”.

### Statistical analysis

The normally distributed data were presented as mean ± standard deviation, the non-normally distributed data were expressed as the median (25, 75% percentile), and categorical data were presented as the number of case (percentages). Normally distributed continuous variables were compared by One-way analysis of variance (ANOVA), non-normally distributed continuous variables were compared by Kruskal-Wallis analysis and categorical variables were compared by the chi-square test or likelihood ratio chi-square test. Indexes with differences in the above monofactor analysis will be included in regression equation. Associated factors of possible sarcopenia and sarcopenia were analysed by multivariate logistic regression analysis with stepwise method, the results expressed as odds ratios (OR) and 95% confidence intervals (CI), α < 0.05 was considered as statistically significant.

## Results

### Prevalence of possible sarcopenia and sarcopenia

Data were included on a total of 729 participants, including 240 males and 489 females aged 60–89 years (mean 67.4 years). Table 3 shows the prevalence of possible sarcopenia and sarcopenia. The prevalence of possible sarcopenia was 11.11,8.33% in men and 12.47% in women respectively. The prevalence of sarcopenia was 10.29, 9.17% in men and 10.84% in women respectively. Either the prevalence of possible sarcopenia or the prevalence of sarcopenia between men and women was not significant difference (*P* = 0.095 & *P* = 0.485, respectively in chi-square test).

**Table 3:** Prevalence of possible sarcopenia and sarcopenia in participants stratified by gender, n(%)

	Overall (*n* = 729)	Men (*n* = 240)	Women (*n* = 489)	*P* value
No-sarcopenia	573(78.60)	198(82.50)	375(76.69)	0.072
Possible sarcopenia	81(11.11)	20(8.33)	61(12.47)	0.095
Sarcopenia	75(10.29)	22(9.17)	53(10.84)	0.485

### Baseline characteristics of participants

The demorgraphic information, lifestyle and clinical characteristics of participants in no-sarcopenia group, possible sarcopenia group and sarcopenia group are summarized in Table 4 (see page 28). There were significant differences among the three groups in terms of age, BMI, body fat percentage, diabetes mellitus, hyperlipidemia, arthritis, number of diseases, scores of MMSE, education background, frequency of alcohol consumption, sedentary time and physical activities level (*P*<0.05).

**Table 4:** Baseline characteristics of participants in three groups

	No-sarcopenic (*n* = 573)	Possible sarcopenia (*n* = 81)	Sarcopenia (*n* = 75)	F/Z/χ2	*P*
Sex				3.614	0.164
male	198 (0.82)	20 (0.08)	22 (0.09)		
female	375 (0.77)	61 (0.13)	53 (0.11)		
Age (years)	66.52 ± 5.52	68.73 ± 5.77	72.96 ± 7.20	44.063	< 0.001
BMI (kg/m^2^)	25.50 ± 3.22	26.04 ± 3.11	21.93 ± 2.64	45.740	< 0.001
Body fat percentage (%)	33.96 ± 7.04	36.02 ± 6.93	31.67 ± 7.49	7.351	0.001
Hypertension				0.823	0.663
Yes	422 (0.79)	56 (0.11)	56 (0.11)		
No	151 (0.77)	25 (0.13)	19 (0.10)		
Resting heart rate (bpm)	68.58 ± 8.70	67.27 ± 8.64	69.93 ± 8.77	1.822	0.162
Diabetes mellitus				9.635	0.008
Yes	82 (0.75)	21 (0.19)	7 (0.06)		
No	491 (0.79)	60 (0.10)	68 (0.11)		
Osteoporosis				0.348	0.840
Yes	52 (0.76)	9 (0.13)	7 (0.10)		
No	521 (0.79)	72 (0.11)	68 (0.10)		
Hyperlipidemia				5.120	0.077
Yes	104 (0.75)	23 (0.17)	12 (0.09)		
No	461 (0.79)	58 (0.10)	63 (0.11)		
Arthritis				8.547	0.014
Yes	67 (0.67)	18 (0.18)	14 (0.14)		
No	506 (0.80)	63 (0.10)	61 (0.10)		
Number of diseases	1 (1,2)	2 (1,4)	1 (0,3)	11.317	0.003
PSQI	9 (5,13)	8 (5,14)	10 (6,15)	1.522	0.467
Score of MMSE	27.48 ± 2.70	27.11 ± 2.80	26.39 ± 3.41	5.373	0.005
Education background				13.965	0.007
Primary school or below	33 (0.69)	8 (0.17)	7 (0.15)		
Secondary school or technical secondary school	473 (0.80)	66 (0.11)	50 (0.09)		
University, college or above	67 (0.73)	7 (0.08)	18 (0.20)		
Smoking status				0.235	0.889
Yes	59 (0.78)	8 (0.10)	9 (0.12)		
No	514 (0.79)	73 (0.11)	66 (0.10)		
Frequency of alcohol consumption				11.598	0.021
More than 3 times/week	52 (0.90)	3 (0.05)	3 (0.05)		
1–3 times/week	426 (0.76)	71 (0.13)	65 (0.12)		
No	95 (0.87)	7 (0.06)	7 (0.06)		
Type of staple food				2.611	0.625
Refined grain	315 (0.80)	50 (0.12)	44 (0.11)		
Refined grain and coarse food grain are equal	206 (0.80)	26 (0.10)	27 (0.10)		
Coarse food grain	52 (0.85)	5 (0.08)	4 (0.07)		
Habit of drinking milk				4.977	0.547
Every day	227 (0.76)	36 (0.12)	37 (0.12)		
4–6 times/week	70 (0.81)	9 (0.10)	7 (0.08)		
1–3 times/week	90 (0.77)	16 (0.14)	11 (0.09)		
No	186 (0.82)	20 (0.09)	20 (0.09)		
Bowel movements				3.564	0.168
at least once a day	486 (0.79)	71 (0.12)	58 (0.09)		
Once every 2 days or above	87 (0.76)	10 (0.09)	17 (0.15)		
Means of transport				2.290	0.318
Relatively high-intensity means of transport	227 (0.79)	28 (0.10)	32 (0.11)		
Relatively low-intensity means of transport	296 (0.76)	53 (0.14)	43 (0.11)		
Sedentary time (minutes)	371.38 ± 99.35	410.74 ± 99.34	423.60 ± 106.92	13.001	< 0.001
Physical activity level				40.553	< 0.001
Low level	51 (0.59)	15 (0.17)	20 (0.23)		
Moderate level	304 (0.76)	54 (0.14)	41 (0.10)		
High level	218 (0.89)	12 (0.05)	14 (0.06)		

### Factors associated to possible sarcopenia and sarcopenia

Eleven indicators with statistical significance in univariate analysis were selected for logistic regression analysis, including age, BMI, body fat percentage, diabetes mellitus, arthritis, number of diseases, score of MMSE, education background, frequency of alcohol consumption, sedentary time and physical activity level associated to possible sarcopenia and sarcopenia with multivariate logistic regression analysis with stepwise method. Finally, the stepwise regression equation chose five factors for analysis, including age, BMI, body fat percentage, MMSE total score and physical activity level. We found that age (OR:1.047, 95%CI:1.005–1.090) and lower physical activity level (low level OR:4.171, 95% CI:1.790–9.720; moderate level OR:2.634, 95% CI:1.352–5.132) were significant associated with possible sarcopenia. Age (OR:1.187, 95%CI:1.124–1.253), low BMI (OR:0.424, 95%CI:0.346–0.519), high body fat percentage (OR:1.225, 95%CI:1.140–1.317), low score of MMSE (OR:0.865, 95%CI:0.781–0.958) and low physical activity level (OR:4.638, 95%CI:1.683–12.782) were risk factors for sarcopenia. The results are presented in Table 5.

**Table 5:** Associated factors of possible sarcopenia and sarcopenia

Group	B	SE	Walds	*P*	OR(95%CI)
Possible sarcopenia					
Age	0.045	0.021	4.779	0.029	1.047(1.055–1.090)
BMI	−0.034	0.053	0.410	0.522	0.966(0.870–1.073)
Body fat percentage	0.044	0.025	3.021	0.082	1.045(0.994–1.097)
MMSE total score	−0.043	0.043	1.014	0.314	0.957(0.880–1.042)
Low physical activity level	1.428	0.432	10.952	0.001	4.171(1.790–9.720)
Moderate physical activity level	0.968	0.340	8.095	0.004	2.634(1.352–5.132)
Sarcopenia					
Age	0.172	0.028	38.313	<0.001	1.187(1.124–1.253)
BMI	−0.859	0.103	69.207	<0.001	0.424(0.346–0.519)
Body fat percentage	0.203	0.037	30.406	<0.001	1.225(1.140–1.317)
MMSE total score	−0.146	0.052	7.801	0.005	0.865(0.781–0.958)
Low physical activity level	1.534	0.517	8.797	0.003	4.638(1.683–12.782)
Moderate physical activity level	0.302	0.397	0.580	0.446	1.353(0.622–2.944)

## Discussion

We found that the prevalence of possible sarcopenia among the community-dwelling participants over 60 years old in Tianjin totally was 11.11, 8.33% in males and 12.47% in females, according to the 2020 national census data, there were as many as more than 3 million old adults over the age of 60 in Tianjin [22]. The screening of possible sarcopenia therefore needs to be paid more attention to early prevention sarcopenia and its negative healthy outcomes. According to AWGS2019 criteria, we found the prevalence of sarcopenia among the community-dwelling participants was 9.17% for males and 10.84% for females, which was similar to the prevalence of AWGS2019 report [5].

Our study shows that ageing is an independent risk factor of possible sarcopenia and sarcopenia, indicating that possible sarcopenia and sarcopenia may occur gradually in physiological characteristics, and neuromuscular function with ageing, followed by degenerative changes in skeletal muscle structure and mass. These results are also consistent with previous studies, human may lose 20–30% of skeletal muscle mass from 20 to 80 [23]. Muscle strength begins to decline around age 30 and declines rapidly around age 50 [24]. With the increase of age, motor unit remodeling increases, the rate of muscle fiber denervation increases, protein synthesis decreases, and the number of muscle satellite cells required for skeletal muscle growth and repair decreases [25–28], this can lead to a decreased in muscle fiber cross-sectional area. Old adults lose more type II muscle fibers than that of type I muscle fibers, and the loss of maximal muscle strength is obvious. Therefore, AWGS2019 and EWGSOP2 recommended that the old adults should be early screened for possible sarcopenia to prevent its development [5, 19].

We found that low physical activity level was associated to possible sarcopenia and sarcopenia. The grip strength of old adults with possible sarcopenia and sarcopenia both decreased significantly, and the lower the level of physical activity, the lower the grip strength [29], the more severe the decline in physical function, and the higher the risk of possible sarcopenia and sarcopenia [30]. Low physical activity level leads to impaired muscle cell metabolic function, resulting in loss of muscle mass and strength, while high physical activity level may maintain or improve muscle strength in old adults by promoting protein synthesis, improving low-level chronic inflammatory states, increasing antioxidant capacity, improving the skeletal muscle cell metabolic function, and increasing mitochondrial numbers [31–33]. Therefore, it is suggested that promoting old adults to actively participate in physical activities in the early stage of old age is very significant to prevent the occurrence and development of sarcopenia, reduce disability, and improve their quality of life.

Cognitive dysfunction is associated with sarcopenia. Skeletal muscle is not only a part of the motor system, but also an endocrine organ. Myokines produced by skeletal muscle contraction play autocrine, paracrine and endocrine effects [34], and are involved in muscle proliferation, differentiation and regeneration [35, 36]. They also mediate signal transduction between muscle and bone, brain, liver, pancreas, adipose tissue, vascular bed, and skin [37]. Although the exact mechanism between cognitive dysfunction and sarcopenia is unclear, current studies have shown that myokines are involved in regulating brain functions, including mood, learning, active movement and protecting nerves from damage, confirming the existence of crosstalk between muscles and the brain [38]. In addition, increase physical activity can delay or even prevent the loss of skeletal muscle mass, maintain or restore cognitive function, and improve the progression of neurological diseases [39]. Studies have shown that patients with sarcopenia have various degrees of cognitive dysfunction that results in reducing executive ability and processing speed, which reduce their activities of daily living, the ability and willingness to participate in exercise, and the ability to follow and implement treatment regimens [40]. Therefore, the prevention of cognitive dysfunction is one of the important means to prevent sarcopenia.

Our study found that higher BMI is a protective factor for sarcopenia, which is consistent with previous findings [11, 41]. The average BMI of the participants with sarcopenia in our study was 21.93 kg/m^2^, which was similar to the result of Landi’s result, who found that old adults with a BMI greater than 21 kg/m^2^ had 0.76 times the risk of sarcopenia compared with those with a BMI of less than 21 kg/m^2^ [41]. Miura [11] reported that the average BMI of 65-year-old Japanese people with sarcopenia was only 19 kg/m^2^, indicating that BMI reduction increases the risk of sarcopenia.

Increased body fat mass was an associated risk factor of sarcopenia in our study, which seems to suggest that only a larger BMI with increased lean body mass is more beneficial in reducing the risk of sarcopenia. BMI is one of the criteria for measuring obesity, and some studies suggest that obesity may reduce the risk of sarcopenia by increasing energy reserves and thus improving survivability [42], however, BMI doesn’t distinguish from various body components such as fat, muscle and bone. Measurement of body fat composition can be used to show in greater detail what kind of body composition increase associated with increased BMI may be beneficial in reducing the risk of sarcopenia. Studies have shown that obesity is one of the important factors leading to sarcopenia. Obesity increases the level of pro-inflammatory factors, promoting insulin resistance and sarcopenia [43]. Ageing and changes in lifestyle, dietary habits and hormone levels lead to changes in body composition [44]. We incorporated body fat percentage into the analysis equation and found that high body fat percentage was associated with sarcopenia, which is also an important finding of this study. Fat mass increases with ageing and can gradually infiltrate into skeletal muscle, resulting in changes in muscle fiber structure and contracting property, leading to loss of skeletal muscle mass, strength and function [45]. The molecular biological mechanism of the increase of intramuscular fat content with ageing is not clear. From the perspective of inflammation theory, adipose tissue can release a large amount of IL-6 and TNF-α, which leads to the occurrence of low-level chronic inflammation, reduces insulin sensitivity, impairs the ability of protein synthesis, promotes protein hydrolysis, and leads to the loss of skeletal muscle mass [46]. In addition, the most abundant stem cells and progenitor cells in skeletal muscle are muscle satellite cells and fibro-adipogenic progenitor cells, the former usually differentiate into skeletal muscle cells, while the latter usually differentiate into adipocytes. Ageing may affect the proliferation and differentiation of muscle satellite cells by changing the cytokine secretion, leading to decreased myogenic differentiation and increasing adipogenic differentiation [47]. Li performed aerobic and strength training for 12 weeks in old adults with osteosarcopenic obesity syndrome, which significantly reduced the body fat mass, increased muscle mass, and improved physical function [48], indicating that increased BMI with increased lean body mass may be a more favorable factor for maintaining and improving physical function and reducing the risk of sarcopenia in older adults.

Sarcopenia is the core pathological basis of frailty which increases the risk of death [49]. When old adults have sarcopenia, their physical functions are already greatly impaired. Therefore, early prevention of sarcopenia is critical to the health. The prevalence and its associated risk factors of possible sarcopenia was just attracted an attention in recent years. Accurate understanding of the associated factors of possible sarcopenia and sarcopenia may lead to more advanced preventive measure of sarcopenia and more meaningful for early prevention of sarcopenia. Our study provides a basis for the prevention of sarcopenia. Improving the early identification of sarcopenia in old adults, and promoting the old adults, especially the young-old elderly, to actively increase the physical activity level, reducing body fat mass, and increasing lean body mass to achieve the purpose of preventing sarcopenia is the important inspiration of our study.

There are still some limitations in this study. This study did not differentiate men and women during the risk factor analysis because chi-square test for the possible sarcopenia and sarcopenia prevalence of male and female showed that no difference between genders was found. It had been reported that gender was not a risk factor of the sarcopenia [50, 51]. Indeed, our results may be attributed to relatively small sample size, the further work need to do in the future. Due to the limited sample size, only several factors were taken into analysis. Increasing the sample size and the underlying factors may provide a more comprehensive interpretation of the factors associated to sarcopenia. Further follow-up studies can be conducted to explore the long-term impact of various associated factors on disease development and physical function in patients with possible sarcopenia and sarcopenia.

## Conclusion

We found the prevalence of possible sarcopenia among the community-dwelling old adults was 11.11%, and the prevalence of sarcopenia was 10.29%. Ageing and lower physical activity level were both the associated factors of possible sarcopenia and sarcopenia. High body fat percentage and low cognitive function increase the risk of sarcopenia, suggesting that promoting old adults to participate in physical activity to reduce body fat mass and improve cognitive function is the key to preventing sarcopenia.

## Data Availability

The datasets used and analysed during the current study are available from the corresponding author on reasonable request.

## Abbreviations

AWGS: Asia Working Group of Sarcopenia
MMSE: Lower mini-mental state examination
OR: Odds ratio
CI: Confidence interval
PSQI: Pittsburgh Sleep Quality Index
BIA: Bioelectrical impedance analysis
ASM: Skeletal muscle mass
ASMI: Skeletal muscle mass index
